# Differential expression of *Scinderin* and *Gelsolin* in gastric cancer and comparison with clinical and morphological characteristics

**DOI:** 10.17179/excli2020-1335

**Published:** 2020-06-05

**Authors:** Toktam Sadat Tavabe Ghavami, Shiva Irani, Reza Mirfakhrai, Reza Shirkoohi

**Affiliations:** 1 Department of Genetics, Islamic Azad University, Tehran, Iran; 2 Department of Biology, Islamic Azad University, Tehran, Iran; 3 Department of Medical Genetics, Shahid Beheshti University of Medical Sciences, Tehran, Iran; 4 Cancer Research Institute, Tehran University of Medical Sciences, Tehran, Iran

**Keywords:** gastric cancer, Gelsolin, Scinderin, expression, up-regulation, down-regulation

## Abstract

Gastric cancer is the first cause of cancer-related death in males and the second in female patients in Iran. Advanced cancer is usually associated with distant metastasis, which is uncontrollable. This study was conducted to compare the expression of *Scinderin* and *Gelsolin* genes between gastric cancer and adjacent normal tissue samples in Iranian patients in order to better understand the role of these genes in this disease and to assess them as potential gastric cancer diagnostic or prognostic biomarkers. This case-control study was conducted in 41 Iranian patients suffering from stage I to IV of Gastric Cancer diagnosed by pathologic and endoscopic tests. In this study, significant down-regulation of *Gelsolin *(p=0.001) and over-expression of *Scinderin* (p=0.001) were observed in tumor tissues compared to the adjacent normal tissues. The results of the present study showed decreased *Gelsolin* expression in patients above 40 years, while the relationship between *Gelsolin* expression and age was not significant; also, a significant increase was observed in *Scinderin* expression in patients above 40 years. Furthermore, Lymph node metastasis was observed in 59.52 % of the cases. The results showed that reduced *Gelsolin* and increased *Scinderin* expression were related to lymph node metastasis. Based on results, a significant association was observed between tumor size and *Scinderin* expression level. Furthermore,* Gelsolin* and *Scinderin* expressions were assessed in different grades and stages to determine the association of this gene with cancer progression. The result indicates significant alteration in *Scinderin* expression level of I and IV, II and IV, and III and IV stages. Although no significant association was observed between *Scinderin* expression level and GC grade, the mean *Gelsolin *expression showed a significant difference between grade II and III as well as grade I and IV. Based on our results, these genes would be potential biomarkers.

## Introduction

Gastric cancer (GC) is one of the most prevalent cancers and a leading cause of oncologic death globally. In spite of recent advances in diagnosis and therapeutics, its mortality and incidence rates are still high due to a delayed diagnosis. Approximately 700,000 GC-related deaths are reported annually (Bertuccio et al., 2009[4]; Siegel et al., 2020[40]). It can be cured by surgery at early stages but most GC patients are detected in advanced and metastasis stages. Consequently, poor metastasis prognosis might hinder this mortality rate (Necula et al., 2019[33]; Sun et al., 2012[42]; Torre et al., 2015[46]).

Early metastasis detection might play a valuable role in decreasing the incidence and mortality rates of the disease. During metastasis, mesenchymal cells in the distant organs, become epithelial cells (mesenchymal-TO-epithelial cells transition: MET) and induces an irregular growth of cancer cells. The epithelial-to-mesenchymal transition (EMT), a pathologic phenomenon in cancer, plays an important role in tumor metastasis and progression (Lee et al., 2006[23]; Shirkoohi, 2013[38]).

EMT occurs during normal embryonic development, organ fibrosis, and tissue regeneration. In the first stage, its promotion will make metastasis a life-threatening stage of cancer while in the second stage, EMT would lead to organogenesis, which is necessary for all living organisms. Another phenomenon occurs during the wound healing process, which can result in fibrosis if it is not properly regulated. The stimuli were the same in two cases, but they were different in one. Understanding the cellular behavior in this process and what makes them invasive resistant stemness cells is very important for highlighting cancer treatment modalities (Ghazimoradi and Farivar, 2020[12]; Shirkoohi, 2013[38]).

Although many studies have been conducted to uncover the mysteries of cancer initiation, progression, and invasion at the molecular level, the exact molecular mechanism has not been fully elucidated yet. So, improving our knowledge of the molecular mechanisms underlying cancers might lead to discovering new diagnostic and therapeutic biomarkers (Lazăr et al., 2016[22]).

Since actin-binding proteins play an essential role in regulating actin polymerization, these proteins have been important in cancer research (Gross, 2013[14]). It has been observed that *Gelsolin* protein (a protein expressed in all mammalian tissues) plays an important role in actin reorganization and remodeling of apoptotic cells (Migocka-Patrza et al., 2019[32]; Shirkoohi et al., 2012[39]). In addition, *Scinderin* protein is one of the actin-binding and separating proteins that regulates the activity of cytoskeletal actin filaments (Trifaro et al., 2000[48]).

*Gelsolin,* which is encoded by the GSN gene, plays a crucial role in normal cell motility and morphology maintenance by severing, capping and nucleating actin filaments (Ditsch and Wegner, 1994[8]; Gremm and Wegner, 2000[13]; Ong et al., 2020[34]; Selden et al., 1998[37]). *Gelsolin* regulates Rac (a member of Rho family) that regulates actin filament organization. It has been shown that silencing *Gelsolin* correlates with Rac up-regulation and an increase in the amounts of F-actin in stress fibers, which may result in lamellipodia (Li et al., 2012[25]; Tanaka et al., 2006[43]; Trejo-Cerro et al., 2019[47]). Aberrant expression of *Gelsolin *has been observed in both human and animal cancer initiation (Asch et al., 1996[3]). Cancer cells may evade the apoptosis signaling pathway through *Gelsolin* down-regulation (Kwiatkowski, 1999[20]). Although a large body of evidence suggests that *Gelsolin* might act as a tumor suppressor gene, its up-regulation enhances metastasis in later cancer stages. Thus, *Gelsolin* plays critical roles in human malignancies from initiation to invasion and metastasis (Huang et al., 2015[17]). Stock et al. (2015[41]) examined the impact of *Gelsolin* on breast cancer cell migration. Moreover, they studied the relationship between this gene expression and metastasis-free survival in node-negative breast cancer. It is notable that over-expression of *Gelsolin* gene in MCF7 and MDA-DB-468 cell lines decrease cancer cells migration and metastasis while its knockdown had reverse impact. Tanaka et al. (2006[43]) reported that the expression of *Gelsolin* was indistinguishable in human mammary epithelial cells. Mielnicki et al. (1999[31]) demonstrated the negative regulation of *Gelsolin* expression in breast cancer during epigenetic mechanisms. It has been stated that over-expression of *Gelsolin*, as a tumor suppressor, prevents the growth of bladder, breast, and lung cancer cells (Sagawa et al., 2003[36]). It is notable that the increased amount of histone deacetylases depends on *Gelsolin* expression suppression in gastric cancer (Kim et al., 2004[19]). The relationship between decreased *Gelsolin* expression and tumor metastasis supports this idea (Li et al., 2009[26]). Shirkoohi et al. (2012[39]) introduced *Gelsolin* as a cytoskeletal tumor suppressor and reported that *Gelsolin* level expression affected cell differentiation. The controversy of *Gelsolin* role might be answered by its relationship with estrogen receptor. Stock et al. (2015[41]) state that patients with breast cancer whom their cancer are ER positive show a correlation among higher *Gelsolin* expression and lower tumor stage, grade, cell proliferation and higher metastasis-free survival rate, however ER negative patients have a converse relationship.

Furthermore*, Scinderin* is a member of the *Gelsolin* supper family that plays a key role in actin filament reconstruction in a Ca^2+^ dependent manner by its three actin-, two PIP2-, and two Ca^2+^-binding sites (Chen et al., 2014[5]; Dumitrescu Pene et al., 2005[10]; Lejen et al., 2002[24]). By controlling F-actin dynamics and cytoskeleton underneath the plasma membrane, *Scinderin* plays a significant role in vesicle translocation and endocytosis (Chen et al., 2014[5]; Dumitrescu Pene et al., 2005[10]). According to observations in different studies, *Scinderin* over-expression has been reported in many malignancies (Liu et al., 2015[28]; Qiao et al., 2018[35]). It has been suggested that *Scinderin* silencing could decrease cells proliferation, induce cancer cell cycle arrest and apoptosis. It is notable that knockdown of *Scinderin* led to the suppression of EGFR expression level. For these reasons, many researchers have devoted their studies on *Scinderin* silencing, as a therapeutic strategy, because it plays an important role in cancer cell growth and development (Chen et al., 2014[5]; Lai et al., 2018[21]; Lin et al., 2019[27]). Chen et al. (2014[5]) studied the effect of *Scinderin* silencing in the SGC-7901 cell line and found a significant decrease in cell migration. It has been reported that *Scinderin* silencing inhibits cell migration, filopodia formation, and cell invasion ability (Dumitrescu Pene et al., 2005[10]; Liu et al., 2016[30]).

The purpose of this study was to investigate the expression levels of *Gelsolin* and *Scinderin* in GC and normal tissue samples. In addition, the relationship between the expression levels of these genes and clinical features was also evaluated in these patients.

## Materials and Methods

### Sample collection

This case-control study was conducted in 41 Iranian patients suffering from stage I to IV of Gastric Cancer diagnosed by pathologic and endoscopic tests in Imam Khomeini Hospital (Tehran, Iran). The Ethics Committee of TUMS approved the project, and informed consent was obtained from all participants (34728). Both tumor and normal tissue samples were obtained from Tumor Bank of Iran, Cancer Institute, Imam Khomeini Hospital Complex, Tehran University of Medical Sciences, snap-frozen in liquid nitrogen immediately, and stored at -80 °C until used for RNA extraction. A histological diagnosis of gastric cancer was an inclusion criterion.

### RNA extraction and cDNA synthesis 

Total RNA was extracted using the RiboEX (Genall, South Korea) RNA extraction kit. DNase1 (Sinaclon, Iran) was used to eliminate DNA contamination. A spectrophotometer was used to estimate RNA concentration. Thermo cDNA synthesis kit was used for cDNA synthesis using 1 µg of total extracted RNA according to the manufacturer's instructions.

### Real-time RT-PCR analysis

To assess the expression level of the genes of interest, quantitative real-time PCR was carried out using Corbett (Rotor-Gene 6000) and SYBR Premix Ex Taq II (Takara). For each sample, the PCR reaction reagent comprised 2 µL cDNA mixed with 1 µL forward and reverse primers and 10 µL SYBR green master mix (Takara, Japan). Then, 7 µL nuclease-free water was added to reach a final volume of 20 µL. The thermal cycling condition included an initial denaturation step at 95 ºC for 15 min, 40 cycles at 95 ºC for 15 s, at 60 ºC for 30 s, and at 72 ºC for 20 s. All reactions were done in triplicate. The data are presented as the fold change in gene expression using the 2-ΔΔCT method and normalized to the ACTB gene. The specific primer sequences for genes included in this study were shown in Table 1(Tab. 1).

### Statistical analysis

SPSS 16.0 (SPSS Inc. Chicago, USA) was used for data analysis. Data distribution was evaluated using the one-sample Kolmogorov-Smirnov test. Independent sample t-test was applied to compare the differences between the two groups. One-way ANOVA was used to analyze the relationships between parameters. Numerical data are presented as mean ± standard deviation (SD). P values less than 0.05 were considered significant. 

## Results

### Demographic and clinical characteristics of the selected patients

Forty-five cases (mean age: 68.5) with GC stages I, II, III and IV were included in this study. Other demographic and clinical characteristics of the patients such as lymph node metastasis and tumor size are summarized in Table 2(Tab. 2). The heat maps of *Scinderin* and *Gelsolin* genes are presented in Figure 2A.

### Analysis of Gelsolin and Scinderin expression levels in tumor and non-tumor samples

**Figure 1:**
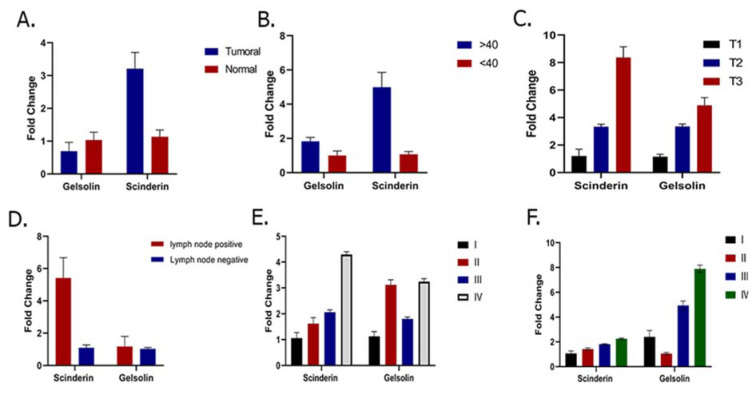
A. Comparison of *Gelsolin *and* Scinderin *expression level in GC patients: this figure demonstrates significant down-regulation of* Gelsolin *and up-regulation of *Scinderin* expression levels in GC in comparison with adjacent normal tissue (p = 0.001). B. Relationship between *Scinderin* and *Gelsolin* expression and patient's age. *Scinderin* expression level was significantly associated with age (p= 0.01). C. Relationship between *Scinderin* and *Gelsolin* and tumor size: D. Relationship between* Scinderin *and* gelsolin *expression and lymph node metastasis. E. Relationship between* Scinderin *and* Gelsolin *expression and different stages of GC. F. Relationship between* Scinderin *and* Gelsolin *expression and different grades of GC.

The results showed a significant down- regulation and up-regulation in the expression levels of* Gelsolin *and* Scinderin *in the GC tumoral tissue compared to the adjacent non-tumoral tissue (*p *value of 0.001), respectively. The expression of *Gelsolin* and* Scinderin *had a 2.38111 ± 0.55671 and -1.39276 ± 0.45563 fold change in GC tumor tissue samples compared to normal tissues, respectively (Figure 1A(Fig. 1)). Meanwhile, the expression of *Gelsolin* was significantly lower and the expression of* Scinderin *was significantly higher in tumor tissues compared to non-tumor tissues obtained from the same patients in this study. The results showed *Gelsolin* down-regulation in 87.7 % and up-regulation in 12.19 % of patients. Moreover, *Scinderin *was up-regulated in 85.38 % and down-regulated in 14.63 % of the patients.

### Relationship between expression level of gelsolin and Scinderin and clinical features in GC patients

As the results, the expression level of *Scinderin* had a significant association with the patent's age (*p* value of 0.01). The age range of the patients with gastric cancer was 33-104 years, which was dependent on their family history and genetic background (see Table 2(Tab. 2)). A significant increase in *Scinderin *up-regulation and *Gelsolin *down-regulation was observed with an increase in age (Figure 1B(Fig. 1))*.*

Tumor size is one of the criteria for tumor growth that is used by pathologists to determine the tumor stage and grade. In this study, tumors were classified into three groups: tumors smaller than 2 cm (T1), tumors between 2 and 5 cm (T2), and tumors larger than 5 cm (T3). According to the results of one-way ANOVA, there was no significant relationship between the *Gelsolin* gene expression level and tumor size while there was a significant relationship between the *Scinderin* expression level and tumor size (p value = 0.02) (Tables 3(Tab. 3) and 4(Tab. 4)). Based on the results, 2.56 % of the tumors were below 2 cm, 58.98 % were 2 to 5 cm, and 38.46 % of tumors were larger than 5 cm in size. Decreased expression of *Gelsolin* and increased expression of *Scinderin* were more evident in larger tumors (Figure 1C(Fig. 1)).

In this study, the patients with gastric cancer were classified by lymph node metastasis, which included 25 patients with lymph node metastasis and 16 patients without it. A correlation was seen between the level of *Scinderin *gene expression and lymph node metastasis, however there was no significant relationship between the level of *Gelsolin *gene expression and lymph node metastasis (p value= 0.005). A sharp increased expression of *Scinderin *and a reduction of expression of *Gelsolin *was more significant in samples with positive lymph node metastasis (60.97 %) in contrast with samples with no lymph node metastasis (39.02 %). There is little likelihood of a link between the expression level of these genes and lymph node metastasis (Figure 1D(Fig. 1)).

Furthermore,* Gelsolin* and *Scinderin* expression was assessed in different grades and stages to determine the association of this gene with cancer progression. The result indicates no significant association between *Gelsolin* expression level and GC stage while *Scinderin* expression level was significantly different between stage I (2.30 ± 2.75) and IV (9.6 ± 6.35) (p= 0.05), II (2.91 ± 2.32) and IV (9.60 ± 6.35) (p=0.005), and III (3.42 ± 3.83) and IV (9.6 ± 6.35) (p=0.005) (Figure 1E(Fig. 1)). 

Although no significant association was observed between *Scinderin* expression level and GC grade, the mean *Gelsolin* expression showed a significant difference between grade II (0.25 ± 0.26) and III (1.01 ± 1.29) (p =0.05) as well as grade I (0.25 ± 0.26) and IV (1.84 ± 3.23) (p=0.04). It is difficult to evaluate the average expression level of *Gelsolin* in grades II and IV and further studies are needed (p=0.06) (Figure 1F(Fig. 1)).

### Evaluation of the biomarker quality for GC

**Figure 2:**
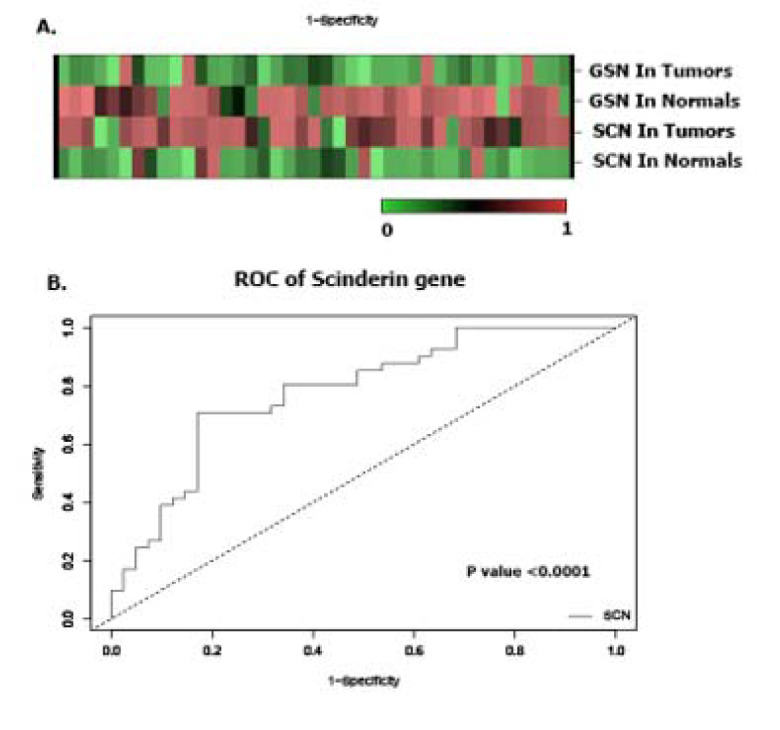
A. The heatmap (Euclidian distance, complete linkage) of *Scinderin* and *Gelsolin* genes. This figure shows high expression of *Scinderin* and *gelsolin* in green (GC) and their low expression in red (normal tissues). B. ROC curve analysis was done to differentiate tumor from non-tumor tissue samples. ROC curve and its calculated area under curve (AUC) of *Scinderin* showed that it might be a suitable tumor biomarker for GC.

ROC curve analysis was used to evaluate the sensitivity and specificity of the expression levels of these genes to differentiate GC tissues from healthy tissues. The calculated levels of the genes examined in this study indicated that *Scinderin* could potentially candidate as an efficient diagnostic biomarker for GC (Figure 2B(Fig. 2)).

## Discussion

Gastric cancer is the third leading cause of cancer-related death in spite of recent advances in diagnostic and therapeutic techniques. Therefore, identification of the molecular mechanisms underlying the disease has drawn the attention of many researchers since it might help to improve cancer therapy and diagnosis (Lazăr et al., 2016[22]; Torre et al., 2015[46]).

Irregular epithelial cell growth and angiogenesis are the first signs of early epithelial cancer (Kalluri and Weinberg, 2009[18]). This process is responsible for the death of more than 90 % of patients with cancer. In many studies, the EMT process has been mentioned as the main mechanism for the formation of malignant phenotypes of epithelial cancer cells (Guo and Giancotti, 2004[15]).

In addition, more evidence suggests that alterations in polymerization or actin remodeling play a key role in regulating phenotypic and morphological events in malignant cells (Thiery and Chopin, 1999[44]). Cancer cell migration can be induced by regulation of the cytoskeleton, since Actin-binding proteins have been investigated in cancer trial (Zhang and Tong, 2010[55]). The expression levels of two important actin-binding proteins, *Gelsolin *and *Scinderin*, were studied in gastric tumor and the adjacent normal tissue in this study.

*Scinderin *may regulate the EMT process to affect cell migration. Chen et al. (2014[5]) reported that *Scinderin* suppression correlated with the proliferation and migration of gastric cancer SGC-7901 cells and attenuated their EMT process. Also, *Gelsolin *is down-regulated in human gastric cancer; moreover, *Gelsolin* significantly correlates with gastric cancer metastasis. Migration of gastric cancer cells is suppressed by *Gelsolin*, which decreases EMT by inducing cytoskeleton remolding via inhibition of p38 signaling (Yuan et al., 2016[54]). All the mentioned studies confirmed the critical role of *Scinderin* and *Gelsolin* in tumorigenesis.

It has been shown that the closest homologue of *Scinderin* is involved in the cell death process in zebrafish. Wang et al. (2014[50]) found *Scinderin* over-expression in prostate tumor. Thus, in order to address the function of *Scinderin* in prostate carcinoma cells, its expression was knocked down in the PC3 cell, a prostate carcinoma cell line, using lentivirus-mediated RNAi. Reduced *Scinderin* expression leads to G0/G1 cell cycle arrest through cell cycle-related genes. Moreover, *Scinderin* silencing inhibits the proliferation and colony formation ability of PC3 cells. Liu et al. (2015[28]) found that knocking down *Scinderin* expression in A549 and H1299 cell lines by lentivirus-mediated RNAi was associated with similar results, including proliferation inhibition and G0/G1 cell cycle arrest. In addition, Wu et al. (2013[52]) reported a significantly lower expression of *Scinderin* in normal ovarian tissue and benign ovarian epithelial tumors versus ovarian epithelial cancer. However, no significant difference was observed in *Scinderin* expression between normal ovarian tissue and benign ovarian epithelial tumor. In another immunohistochemical study of *Scinderin* expression, Hasmim et al. (2013[16]) reported the over-expression of this protein in head and neck cancer. In line with previous studies, the present study showed significant over-expression of *Scinderin* in gastric tumor tissues compared to the adjacent normal tissue.

Knock out of *Gelsolin *expression as a tumor suppressor gene has been observed in at least three animal species and a variety of etiologies. Dong et al. (2002[9]) showed that reduced expression of *Gelsolin* was related to epigenetic factors and was not due to mutation. Furthermore, Ahn et al. (2003[2]) found that decreased *Gelsolin* expression was one of the most common molecular defects in breast carcinoma. In addition, according to a study by Gay et al. (2008[11]) *gelsolin* down-regulation is an early and constant event in clonal carcinogenesis and can cause adenoma to carcinoma, which confirms previous observations. Liu et al. (2007[29]) reported that concurrent *Gelsolin* and Egr-1 down-regulation might be an important marker for breast cancer. Furthermore, in line with Liu et al. (2007[29]), Winston et al. (2001[51]) also found that *Gelsolin* down-regulation correlated with breast carcinoma progression and might be considered as a diagnostic biomarker for this disease. Similarly, Tanaka et al. (2006[43]) suggested the *Gelsolin *controlled E- and N-cadherin conversion; they demonstrated that *Gelsolin* knock-down led to epithelial-mesenchymal transition in human mammary epithelial cells and might be involved in human mammary tumors development. Shirkoohi et al. (2012[39]) found that monocytic differentiation could be induced in U937 leukemia cells by *Gelsolin* through activation of p21CIP1. Evidence also suggests that up-regulation of *Gelsolin* can inhibit proliferation of colon, bladder, and lung cancer cells (Sagawa et al., 2003[36]; Tsai et al., 2012[49]). Thor et al. (2001[45]) reported that *Gelsolin* was a negative prognostic factor and motility effectors in erbB-2 positive and EGFR positive breast cancer cases. Crowley et al. (2000[6]) found that *Gelsolin* played a key role in ductal morphogenesis in the mouse mammary gland. According to the results of a study by Deng et al. (2015[7]) proliferation, apoptosis, migration, and invasion of human oral carcinoma cells can be regulated by *Gelsolin* over-expression. Contradictory results were observed in the current research since suppression of *Gelsolin* expression correlated with cancer onset, progression, and invasion. 

The results of this study showed no significant association between GC stage and *Gelsolin* expression level. In spite of these results, Yuan et al. (2016[54]) found that *Gelsolin* expression was down-regulated in advanced stages of gastric cancer, including III and IV, in comparison to earlier stages, i.e. I and II. On the other hand, Tsai et al. (2012[49]) reported *Gelsolin* over-expression in late stages of gastric cancer compared to earlier stages. This difference might be due to the limited number of samples in their study. Thus, in order to obtain a more confident conclusion, more extensive studies with larger sample sizes are required. In addition, *Gelsolin* and *Scinderin* expression levels showed a significant association with GC stage. As mentioned earlier, Liu et al. (2016[30]) found no significant association between *Scinderin* expression level and GC grade. However, evidence suggests a significant association between *Scinderin* expression level and ovarian cancer stage (Wu et al., 2013[52]). There was a significant association between* Gelsolin* expression level and GC grade in grades I and II. Nevertheless, more extensive studies with larger sample sizes are needed to assess any possible association between the expression level of this gene and tumor grade in grades II and IV. Winston et al. (2001[51]) found a significant reduction in *Gelsolin* expression level in advanced grades of breast cancer and Yamaguchi and Condeelis (2007[53]) reported a significant association between *Gelsolin* expression level and tumor grade. Liu et al. (2016[30]) found that tumors involving *Scinderin* repression were significantly smaller compared to the tumors expressing this gene. Similar results were also obtained in this study, and a significant association was observed between tumor size and *Scinderin* expression level. In contrast to *Scinderin*, no signification association was found between *Gelsolin* expression level and tumor size. Li et al. (2009[26]) also reported no significant association between *Gelsolin* expression level and tumor size. A significant increase was observed in *Scinderin* expression in patients above 40 years. Nonetheless, Wu et al. (2013[52]) found no significant relationship between *Scinderin* expression and patient's age. The results of the present study showed decreased *Gelsolin* expression in patients above 40 years, while the relationship between *Gelsolin *expression and age was not significant. In two other studies, Winston et al. (2001[51]) and Abedini et al. (2014[1]) also found no significant relationship between *Gelsolin* expression and patient's age. Lymph node metastasis was observed in 59.52 % of the cases included in this study. The results showed that reduced *Gelsolin* and increased *Scinderin* expression were related to lymph node metastasis. However, in order to elucidate their role in lymph node metastasis, more research is warranted into the regulatory pathways of these genes.

## Conclusion

*Scinderin* and *Gelsolin *genes play key roles in gastric cancer initiation, progression, and invasion. According to the result of this study, their expression level significantly correlates with GC. Overall, more extensive *in vitro* and *in vivo* studies with larger sample sizes are warranted to deepen our understanding of the roles of *Gelsolin* and *Scinderin* genes. Moreover, assessment of *Gelsolin* and *Scinderin* expression at the protein level might also be helpful. So, further studies would be promising to find new more efficient biomarkers.

## Conflict of interest

The authors declare that they have no conflict of interests.

## Acknowledgements

We humbly thank Dr Babak Saedi from Tehran University for his help in editing this article.

## Figures and Tables

**Table 1:**
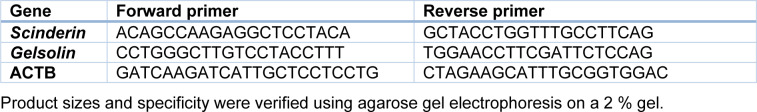
Specific primer sequences

**Table 2:**
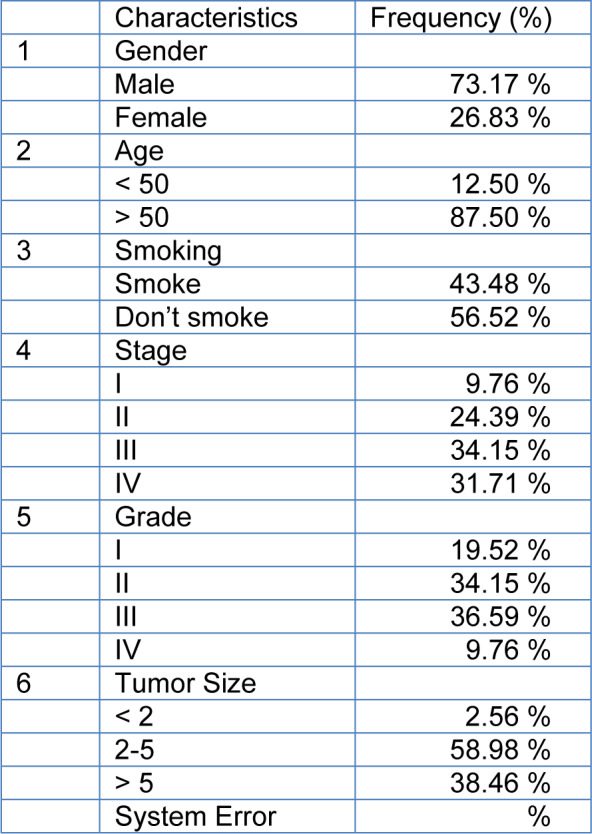
Demographic and clinical characteristics of the selected patients

**Table 3:**
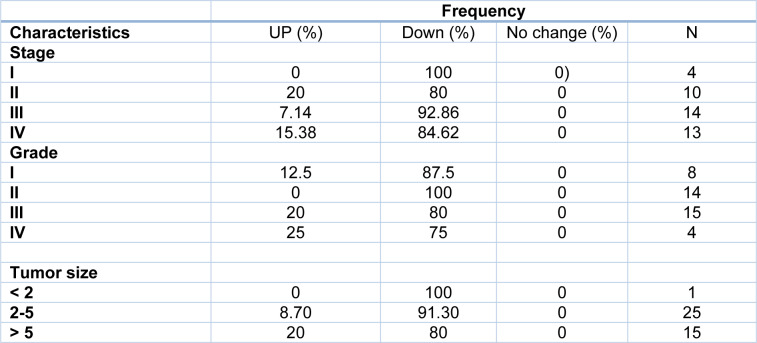
*Gelsolin* expression level and patients' demographical information

**Table 4:**
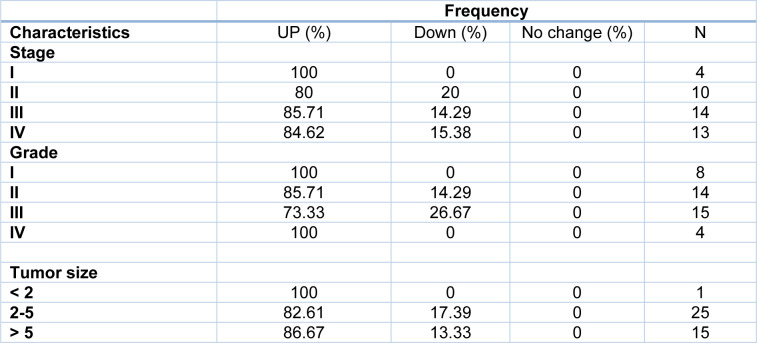
*Scinderin* expression level and patients' demographical information

## References

[1] Abedini MR, Wang PW, Huang YF, Cao M, Chou CY, Shieh DB (2014). Cell fate regulation by gelsolin in human gynecologic cancers. Proc Natl Acad Sci.

[2] Ahn JS, Jang IS, Kim DI, Cho KA, Park YH, Kim K (2003). Aging-associated increase of gelsolin for apoptosis resistance. Biochem Biophys Res Commun.

[3] Asch HL, Head K, Dong Y, Natoli F, Winston JS, Connolly JL (1996). Widespread loss of gelsolin in breast cancers of humans, mice, and rats. Cancer Res.

[4] Bertuccio P, Chatenoud L, Levi F, Praud D, Ferlay J, Negri E (2009). Recent patterns in gastric cancer: a global overview. Int J Cancer.

[5] Chen XM, Guo JM, Chen P, Mao LG, Feng WY, Le DH (2014). Suppression of scinderin modulates epithelial‑mesenchymal transition markers in highly metastatic gastric cancer cell line SGC‑7901. Mol Med Report.

[6] Crowley MR, Head KL, Kwiatkowski DJ, Asch HL, Asch BB (2000). The mouse mammary gland requires the actin-binding protein gelsolin for proper ductal morphogenesis. Dev Biol.

[7] Deng R, Hao J, Han W, Ni Y, Huang X, Hu Q (2015). Gelsolin regulates proliferation, apoptosis, migration and invasion in human oral carcinoma cells. Oncol Lett.

[8] Ditsch A, Wegner A (1994). Nucleation of actin polymerization by gelsolin. Eur J Biochem.

[9] Dong Y, Asch HL, Ying A, Asch BB (2002). Molecular mechanism of transcriptional repression of gelsolin in human breast cancer cells. Exp Cell Res.

[10] Dumitrescu Pene T, Rosé SD, Lejen T, Marcu MG, Trifaró JM (2005). Expression of various scinderin domains in chromaffin cells indicates that this protein acts as a molecular switch in the control of actin filament dynamics and exocytosis. J Neurochem.

[11] Gay F, Estornes Y, Saurin JC, Joly-Pharaboz MO, Friederich E, Scoazec JY (2008). In colon carcinogenesis, the cytoskeletal protein gelsolin is down-regulated during the transition from adenoma to carcinoma. Hum Pathol.

[12] Ghazimoradi MH, Farivar S (2020). The role of DNA demethylation in induction of stem cells. Prog Biophys Mol Biol.

[13] Gremm D, Wegner A (2000). Gelsolin as a calcium‐regulated actin filament‐capping protein. Eur J Biochem.

[14] Gross SR (2013). Actin binding proteins: their ups and downs in metastatic life. Cell Adh Migr.

[15] Guo W, Giancotti FG (2004). Integrin signalling during tumour progression. Nat Rev Mol Cell Biol.

[16] Hasmim M, Badoual C, Vielh P, Drusch F, Marty V, Laplanche A (2013). Expression of EPHRIN-A1, SCINDERIN and MHC class I molecules in head and neck cancers and relationship with the prognostic value of intratumoral CD8+ T cells. BMC Cancer.

[17] Huang GW, Liao LD, Li EM, Xu LY (2015). siRNA induces gelsolin gene transcription activation in human esophageal cancer cell. Sci Rep.

[18] Kalluri R, Weinberg RA (2009). The basics of epithelial-mesenchymal transition. J Clin Invest.

[19] Kim JH, Choi YK, Kwon HJ, Yang HK, Choi JH, Kim DY (2004). Downregulation of gelsolin and retinoic acid receptor β expression in gastric cancer tissues through histone deacetylase 1. J Gastroenterol Hepatol.

[20] Kwiatkowski DJ (1999). Functions of gelsolin: motility, signaling, apoptosis, cancer. Curr Opin Cell Biol.

[21] Lai X, Su W, Zhao H, Yang S, Zeng T, Wu W (2018). Loss of scinderin decreased expression of epidermal growth factor receptor and promoted apoptosis of castration‐resistant prostate cancer cells. FEBS Open Bio.

[22] Lazăr DC, Tăban S, Cornianu M, Faur A, Goldiş A (2016). New advances in targeted gastric cancer treatment. World J Gastroenterol.

[23] Lee JM, Dedhar S, Kalluri R, Thompson EW (2006). The epithelial–mesenchymal transition: new insights in signaling, development, and disease. J Cell Biol.

[24] Lejen T, Pene TD, Rosé S, Trifaró JM (2002). The role of different scinderin domains in the control of F‐actin cytoskeleton during exocytosis. Ann N Y Acad Sci.

[25] Li GH, Arora PD, Chen Y, McCulloch CA, Liu P (2012). Multifunctional roles of gelsolin in health and diseases. Med Res Rev.

[26] Li R, Chen WC, Pang XQ, Hua C, Li L, Zhang XG (2009). Expression of CD40 and CD40L in gastric cancer tissue and its clinical significance. Int J Mol Sci.

[27] Lin Q, Li J, Zhu D, Niu Z, Pan X, Xu P (2019). Aberrant scinderin expression correlates with liver metastasis and poor prognosis in colorectal cancer. Front Pharmacol.

[28] Liu H, Shi D, Liu T, Yu Z, Zhou C (2015). Lentivirus-mediated silencing of SCIN inhibits proliferation of human lung carcinoma cells. Gene.

[29] Liu J, Liu YG, Huang R, Yao C, Li S, Yang W (2007). Concurrent down-regulation of Egr-1 and gelsolin in the majority of human breast cancer cells. Cancer Genom Proteom.

[30] Liu JJ, Liu JY, Chen J, Wu YX, Yan P, Ji CD (2016). Scinderin promotes the invasion and metastasis of gastric cancer cells and predicts the outcome of patients. Cancer Lett.

[31] Mielnicki LM, Ying AM, Head KL, Asch HL, Asch BB (1999). Epigenetic regulation of gelsolin expression in human breast cancer cells. Exp Cell Res.

[32] Migocka-Patrza M, Joanna N, Tarnowska AG, Dubińska-Magiera M, Daczewska MG (2019). Gelsolin and related proteins in vertebrate model organisms. Folia Biol (Krakow).

[33] Necula L, Matei L, Dragu D, Neagu AI, Mambet C, Nedeianu S (2019). Recent advances in gastric cancer early diagnosis. World J Gastroenterol.

[34] Ong MS, Deng S, Halim CE, Cai W, Tan TZ, Huang RYJ (2020). Cytoskeletal proteins in cancer and intracellular stress: a therapeutic perspective. Cancers (Basel).

[35] Qiao X, Zhou Y, Xie W, Wang Y, Zhang Y, Tian T (2018). Scinderin is a novel transcriptional target of BRMS1 involved in regulation of hepatocellular carcinoma cell apoptosis. Am J Cancer Res.

[36] Sagawa N, Fujita H, Banno Y, Nozawa Y, Katoh H, Kuzumaki N (2003). Gelsolin suppresses tumorigenicity through inhibiting PKC activation in a human lung cancer cell line, PC10. Br J Cancer.

[37] Selden LA, Kinosian HJ, Newman J, Lincoln B, Hurwitz C, Gershman LC (1998). Severing of F-actin by the amino-terminal half of gelsolin suggests internal cooperativity in gelsolin. Biophys J.

[38] Shirkoohi R (2013). Epithelial mesenchymal transition from a natural gestational orchestration to a bizarre cancer disturbance. Cancer Sci.

[39] Shirkoohi R, Fujita H, Darmanin S, Takimoto M (2012). Gelsolin induces promonocytic leukemia differentiation accompanied by upregulation of p21CIP1. Asian Pac J Cancer Prev.

[40] Siegel RL, Miller KD, Jemal A (2020). Cancer statistics, 2020. CA Cancer J Clin.

[41] Stock AM, Klee F, Edlund K, Grinberg M, Hammad S, Marchan R (2015). Gelsolin is associated with longer metastasis-free survival and reduced cell migration in estrogen receptor-positive breast cancer. Anticancer Res.

[42] Sun Z, Wang ZN, Zhu Z, Xu YY, Xu Y, Huang BJ (2012). Evaluation of the seventh edition of American Joint Committee on Cancer TNM staging system for gastric cancer: results from a Chinese monoinstitutional study. Ann Surg Oncol.

[43] Tanaka H, Shirkoohi R, Nakagawa K, Qiao H, Fujita H, Okada F (2006). siRNA gelsolin knockdown induces epithelial‐mesenchymal transition with a cadherin switch in human mammary epithelial cells. Int J Cancer.

[44] Thiery JP, Chopin D (1999). Epithelial cell plasticity in development and tumor progression. Cancer Metastasis Rev.

[45] Thor AD, Edgerton SM, Liu S, Moore DH, Kwiatkowski DJ (2001). Gelsolin as a negative prognostic factor and effector of motility in erbB-2-positive epidermal growth factor receptor-positive breast cancers. Clin Cancer Res.

[46] Torre LA, Bray F, Siegel RL, Ferlay J, Lortet‐Tieulent J, Jemal A (2015). Global cancer statistics, 2012. CA Cancer J Clin.

[47] Trejo-Cerro O, Aguilar-Hernández N, Silva-Ayala D, López S, Arias CF (2019). The actin cytoskeleton is important for rotavirus internalization and RNA genome replication. Virus Res.

[48] Trifaro J, Rose S, Marcu M (2000). Scinderin, a Ca2+-dependent actin filament severing protein that controls cortical actin network dynamics during secretion. Neurochem Res.

[49] Tsai MH, Wu CC, Peng PH, Liang Y, Hsiao YC, Chien KY (2012). Identification of secretory gelsolin as a plasma biomarker associated with distant organ metastasis of colorectal cancer. J Mol Med.

[50] Wang D, Sun SQ, Yu YH, Wu WZ, Yang SL, Tan JM (2014). Suppression of SCIN inhibits human prostate cancer cell proliferation and induces G0/G1 phase arrest. Int J Oncol.

[51] Winston JS, Asch HL, Zhang PJ, Edge SB, Hyland A, Asch BB (2001). Downregulation of gelsolin correlates with the progression to breast carcinoma. Breast Cancer Res Treat.

[52] Wu S, Ma J, Jiang Y (2013). The expression of scinderin in epithelial ovarian cancer and the clinicopathologic significance. J Pract Obstet Gynecol.

[53] Yamaguchi H, Condeelis J (2007). Regulation of the actin cytoskeleton in cancer cell migration and invasion. Biochim Biophys Acta.

[54] Yuan X, Wang W, Li J, Zheng P, Dong P, Chen L (2016). Gelsolin suppresses gastric cancer metastasis through inhibition of PKR-p38 signaling. Oncotarget.

[55] Zhang Y, Tong X (2010). Expression of the actin-binding proteins indicates that cofilin and fascin are related to breast tumour size. J Int Med Res.

